# P-350. Epidemiology of *Candida auris* isolated from blood cultures at a tertiary care facility in metro Detroit

**DOI:** 10.1093/ofid/ofae631.551

**Published:** 2025-01-29

**Authors:** Marco R Scipione, Jing Zhao, Lavina Jabbo, Hossein Salimnia, Teena Chopra

**Affiliations:** Detroit Receiving Hospital, Detroit, Michigan; Detroit Medical Center-Harper Hospital, Detroit, Michigan; Department of Infection Control, Detroit Medical Center, Detroit, MI, USA, Detroit, Michigan; Wayne State University, Detroit, MI; Detroit Medical Center, Wayne State University, Detroit, MI

## Abstract

**Background:**

*Candida auris* is a serious global health threat and the number of cases has increased recently. *C. auris i*s often resistant to commonly used antifungals, making it difficult to treat. The objective of this study is to describe the epidemiology of *C. auris* isolated from blood cultures at a tertiary care facility in metro Detroit.

**Figure:**
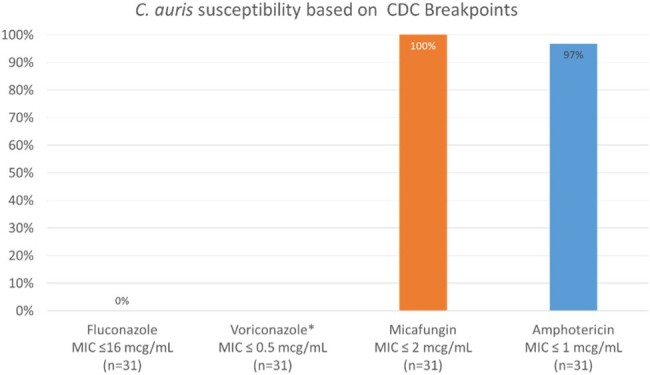

**Methods:**

All *C. auris* isolates from blood cultures at the Detroit Medical Center from 8/1/2019 to 4/25/2024 were reviewed. Patient characteristics as well as microbiological characteristics were evaluated. There are no established susceptibility breakpoints for *C. auris* but the CDC has published tentative MIC breakpoints (mcg/mL) based on closely related Candida species and expert opinion.

**Figure:**
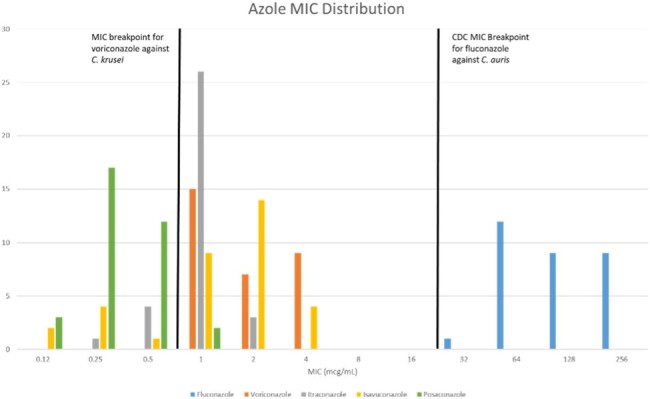

**Results:**

Thirty-eight patients had a positive blood culture with *C. auris*. The median age was 66 years (IQR 60-72). Length of stay was 37 days (IQR 22-62) and length of stay prior to positive blood culture was 25 days (IQR 11-43). Twenty-one (55%) patients had a *C. auris* isolation flag in the electronic medical record (EMR) prior to the positive blood culture. Only 31 patients had susceptibility testing done. All 31 isolates that were tested were resistant to fluconazole (MIC ≥ 32 mcg/mL) and all were susceptible to micafungin (MIC ≤ 2 mcg/mL). Thirty of 31 (97%) isolates were also susceptible to amphotericin B (MIC ≤ 1 mcg/mL). The antifungals with no published breakpoints had the following median MICs: voriconazole 2 mcg/mL (IQR 1-4); itraconazole 1 mcg/mL (IQR 1-1); isavuconazole 2 mcg/mL (IQR 1-2); posaconazole 0.25 mcg/mL (IQR 0.25-0.5); caspofungin 0.25 mcg/mL (IQR 0.15-0.5); anidulafungin 1 mcg/mL (0.5-1). The distribution of MICs for each agents are shown in figure 2 and figure 3.

**Figure:**
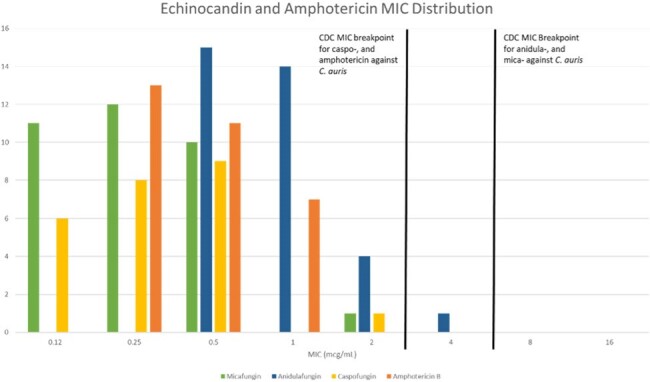

**Conclusion:**

Data are lacking to determine the most appropriate therapy for *C. auris* but based on local epidemiology, the use of an echinocandin as a first-line option for *C. auris* infections is appropriate.

**Disclosures:**

**All Authors**: No reported disclosures

